# COVID-19 Incidence and Mortality Among American Indian/Alaska Native and White Persons — Montana, March 13–November 30, 2020

**DOI:** 10.15585/mmwr.mm7014a2

**Published:** 2021-04-09

**Authors:** Laura L. Williamson, Todd S. Harwell, Todd M. Koch, Stacey L. Anderson, Magdalena K. Scott, James S. Murphy, Greg S. Holzman, Helen F Tesfai

**Affiliations:** ^1^Montana Department of Public Health and Human Services; ^2^Rocky Mountain Tribal Leaders Council, Epidemiology Center, Billings, Montana.

Geographic differences in infectious disease mortality rates have been observed among American Indian or Alaska Native (AI/AN) persons in the United States (*1*), and aggregate analyses of data from selected U.S. states indicate that COVID-19 incidence and mortality are higher among AI/AN persons than they are among White persons (*2*,*3*). State-level data could be used to identify disparities and guide local efforts to reduce COVID-19–associated incidence and mortality; however, such data are limited. Reports of laboratory-confirmed COVID-19 cases and COVID-19–associated deaths reported to the Montana Department of Public Health and Human Services (MDPHHS) were analyzed to describe COVID-19 incidence, mortality, and case-fatality rates among AI/AN persons compared with those among White persons. During March–November 2020 in Montana, the estimated cumulative COVID-19 incidence among AI/AN persons (9,064 cases per 100,000) was 2.2 times that among White persons (4,033 cases per 100,000).* During the same period, the cumulative COVID-19 mortality rate among AI/AN persons (267 deaths per 100,000) was 3.8 times that among White persons (71 deaths per 100,000). The AI/AN COVID-19 case-fatality rate (29.4 deaths per 1,000 COVID-19 cases) was 1.7 times the rate in White persons (17.0 deaths per 1,000). State-level surveillance findings can help in developing state and tribal COVID-19 vaccine allocation strategies and assist in local implementation of culturally appropriate public health measures that might help reduce COVID-19 incidence and mortality in AI/AN communities.

Reports of COVID-19 cases and COVID-19–associated deaths were analyzed to assess the number, percentage, and crude rates of COVID-19 cases and deaths occurring among AI/AN persons and White persons in Montana during March 13–November 30, 2020. Case data were derived from the Montana Infectious Disease Information System. Montana residents who met the definition of a confirmed case (i.e., having received a positive test result for SARS-CoV-2, the virus that causes COVID-19, from a respiratory specimen, using a molecular amplification test and reported to MDPHHS) were included in the analysis. COVID-19–associated deaths were identified from death certificates reported to the MDPHHS Office of Vital Records; COVID‐19 deaths were identified by using *International Classification of Diseases, Tenth Revision* code U07.1, listed as either the underlying or a contributing cause of death. This activity was reviewed by MDPHHS and was conducted for public health surveillance purposes and consistent with applicable state and federal law.^†^

Information on race was available for 49,426 (78%) of 63,339 persons who had received a diagnosis of COVID-19 and for 903 (100%) COVID-19–associated deaths reported to MDPHHS. Persons of AI/AN race were defined as those whose race was reported as AI/AN alone or in combination with other races. Persons of White race were defined as those whose race was reported as White with no other race selected. Ethnicity was not included in this analysis because data on ethnicity was missing for 37% of reported cases. The 2019 National Center for Health Statistics bridged-race population estimates for AI/AN and White persons in Montana were used as denominators to calculate crude cumulative incidence (cumulative cases per 100,000 population) and cumulative mortality rates (cumulative deaths per 100,000 population).^§^^,^^¶^ These population estimates were used to determine that 90.6% of Montana residents were White and that 7.3% were AI/AN. All rates were calculated separately for AI/AN and White persons, overall and by sex and age group. Age group was assessed both categorically (<65 and ≥65 years) and by using medians with interquartile ranges (IQRs). Rate ratios (COVID-19 cumulative incidence and mortality rates among AI/AN persons divided by corresponding rates among White persons) and case-fatality rates (the number of COVID-19–associated deaths per 1,000 reported COVID-19 cases) were also calculated. Corresponding 95% confidence intervals (CIs) were calculated using the Poisson Exact method (*4*). Analyses were conducted using SPSS (version 23; IBM) for incidence estimates and SAS (version 9.4; SAS Institute) for mortality estimates.

During March 13–November 30, 2020, among 49,426 persons in Montana who had received a diagnosis of COVID-19 and for whom information on race was available, 7,069 (14.3%) were AI/AN, and 39,040 (79.0%) were White. The estimated cumulative incidence among AI/AN persons (9,064 cases per 100,000) was 2.2 times the rate among White persons (4,033) (Table). The estimated cumulative incidence was also higher among AI/AN persons than that among White persons by sex and age group. Among AI/AN persons, the estimated cumulative incidence was higher among persons aged ≥65 years (10,321 per 100,000) compared with that among persons aged <65 years (8,947).** Among White persons, incidence was higher among persons aged <65 years (4,137) than among those aged ≥65 years (3,632). The median age of AI/AN persons with COVID-19 was 34 years (IQR = 20–51 years) compared with 42 years (IQR = 26–60 years) among White persons. Among both AI/AN and White persons, estimated cumulative incidence by race was higher among women (9,517 and 4,272 per 100,000, respectively) than among men (8,405 and 3,687, respectively).

**TABLE:** COVID-19 incidence and mortality rates* among American Indian or Alaska Native (AI/AN) and White persons,^†^^,^^§^ by age group and sex**^¶^** — Montana, March 13–November 30, 2020

Characteristic	AI/AN		White		AI/AN to White rate ratio (95% CI)
	No. (%)	Rate (95% CI)	No. (%)	Rate (95% CI)	
**Cumulative incidence**					
**Total**	**7,069 (100)**	**9,064 (8,852–9,275)**	**39,040 (100)**	**4,033 (3,993–4,073)**	**2.2 (2.1–2.5)**
**Sex**					
Female	3,752 (53)	9,517 (9,212–9,821)	20,498 (52)	4,272 (4,213–4,330)	2.2 (2.1–2.4)
Male	3,242 (46)	8,405 (8,116–8,695)	17,995 (46)	3,687 (2,633–3,741)	2.3 (2.1–2.5)
**Age group, yrs**					
<65	6,388 (90)	8,947 (8,728–9,167)	31,842 (82)	4,137 (4,091–4,182)	2.2 (2.0–2.4)
≥65	681 (10)	10,321 (9,546–1,097)	7,198 (18)	3,632 (3,549–3,716)	2.8 (2.6–3.1)
**Cumulative mortality**					
**Total**	**208 (100)**	**267 (232–306)**	**664 (100)**	**71 (66–77)**	**3.8 (3.2–4.4)**
**Sex**					
Female	88 (42)	223 (179–275)	306 (46)	66 (59–74)	3.4 (2.7–4.3)
Male	120 (58)	311 (258–372)	358 (54)	76 (68–84)	4.1 (3.3–5.0)
**Age group, yrs**					
<65	87 (42)	122 (98–150)	72 (11)	10 (8–12)	12.5 (9.1–17.1)
≥65	121 (58)	1,834 (1,522–2,191)	592 (89)	302 (278–328)	6.1 (5.0–7.4)

**Abbreviation:** CI = confidence interval.

* The number of COVID-19 cases or deaths by race and by race and sex/age group per 100,000 in the same race or race and sex/age group.

^†^ Includes Hispanic and non-Hispanic persons.

^§^ Race data were missing for 13,913 of 63,339 (22%) patients, and ethnicity data were missing for 23,435 of 63,339 (37%) patients; race and ethnicity data were complete for all deaths.

^¶^ Sex data were missing for 75 (1%) AI/AN patients and for 547 (1%) White patients.

During March 13–November 30, 2020, among 903 COVID-19–associated deaths in Montana, 208 (23.0%) occurred among AI/AN persons compared with 664 (73.5%) among White persons. The cumulative COVID-19 mortality rate among AI/AN persons (267 deaths per 100,000) was 3.8 times the rate among White persons (71 deaths per 100,000). Cumulative mortality was also higher among AI/AN persons than among White persons by sex and age group. The median age of death among AI/AN persons who died from COVID-19 was 68 years (IQR = 58–75) compared with 82 years (IQR = 73–89) among White persons. The case-fatality rate among AI/AN persons (29.4 deaths per 1,000 COVID-19 cases) was 1.7 times (95% CI = 1.7–1.8) the rate among White persons (17.0 deaths per 1,000 COVID-19 cases).

## Discussion

During March 13–November 30, 2020, COVID-19 incidence and mortality among AI/AN persons in Montana were approximately twice and nearly four times those among White persons, respectively. In addition, the case-fatality rate among AI/AN persons was close to twice that among White persons. Several factors might have contributed to the higher COVID-19 incidence and mortality among AI/AN persons. AI/AN communities in Montana have higher levels of social vulnerability, including living in shared housing, challenges accessing health care and transportation, and lower household incomes.^††^ As well, AI/AN persons might be more likely than White persons to live in multigenerational households or be unable to work from home because of the nature of their work (e.g., being frontline workers) or because they are not able to telework due to the lack of Internet access, which might increase the risk for SARS-CoV-2 infection (*5*). AI/AN persons in Montana also have a high prevalence of chronic health conditions and risk factors for severe illness from COVID-19, including heart disease, type 2 diabetes mellitus, and cigarette smoking.^§§^

An assessment of the impact of COVID-19 among AI/AN persons from 23 states made early during the pandemic (January–July 2020) found that the cumulative incidence rate ratio between AI/AN and White persons was 3.5 (*2*), similar to the current study’s finding. In addition, a study comparing age-adjusted COVID-19 mortality rates among AI/AN and White persons in 14 states during January–June 2020 reported a mortality rate ratio of 1.8 for AI/AN persons compared with that for White persons (*3*), which was lower than that identified in a supplementary analysis conducted by MDPHHS.^¶¶^

The findings in this report are subject to at least three limitations. First, the case-level surveillance and death certificate data might not have been complete at the time of the analysis and are subject to change. Therefore, this analysis likely underestimated the number of persons who had received a diagnosis of COVID-19 and the number of deaths that occurred, particularly more recently. Second, information on race and ethnicity was missing for 22% and 37% of cases, respectively. Because of the large proportion of COVID-19 cases with missing ethnicity information, ethnicity was not included in the analyses. Therefore, COVID-19 patients and decedents whose race was listed as White include some persons whose ethnicity is Hispanic or non-Hispanic. Previous reports have documented that Hispanic populations have disproportionality higher COVID-19 mortality compared with White populations (*6*). However, the extent of bias introduced as a result of this limitation is expected to be minimal because Hispanic persons represent a small proportion of Montana’s population (4.1%). Finally, previous studies suggest that AI/AN persons might be misclassified as non–AI/AN races and ethnicities. However, studies conducted in Montana and northern plains states indicate that race misclassification is relatively less common in these states than it is in other areas of the United States (*7*,*8*).

Understanding the higher COVID-19 incidence, mortality, and case-fatality rates among AI/AN persons can help develop state and tribal COVID-19 vaccine allocation strategies, including adapting the current interim CDC Advisory Committee on Immunization Practices’ COVID-19 vaccine allocation recommendations to prioritize persons at increased risk for poor outcomes or at high risk for exposure to SARS-CoV-2 in AI/AN communities, such as tribal elders, persons living in multigenerational or congregate households, and persons with high-risk medical conditions (*9*). These findings also reinforce the importance of using state-level surveillance to identify disparities among AI/AN or other minority communities to help develop local implementation of culturally informed public health measures and enhanced community education to prevent or limit community transmission of SARS-CoV-2 (*10*).

**Summary**

**What is already known about this topic?**

Aggregate analyses of data from selected U.S. states indicate that COVID-19 incidence and mortality are higher among American Indian or Alaska Native (AI/AN) persons than they are among White persons.

**What is added by this report?**

COVID-19 incidence and mortality rates among AI/AN persons in Montana were 2.2 and 3.8 times, respectively, those among White persons. The case-fatality rate among AI/AN persons was 1.7 times that among White persons.

**What are the implications for public health practice?**

These findings reinforce importance of using state-level surveillance to develop state and tribal COVID-19 vaccine allocation strategies and to inform local implementation of culturally appropriate public health measures that might help reduce COVID-19 incidence and mortality in AI/AN communities.

## Footnotes

* Race data were missing for 13,913 of 63,339 (22%) patients, and ethnicity data were missing for 23,435 of 63,339 (37%) patients. Race and ethnicity data were complete for all deaths. Cumulative incidences by race were considered estimates because of the large proportion of missing race/ethnicity data.

† Mont. Code Ann. Sect. 50–15–122 and 50–16–103; 45 C.F.R. part 46.102(l)(2).

§
https://www.cdc.gov/nchs/nvss/bridged_race/data_documentation.htm

¶ The number of COVID-19 cases or deaths by race and by race and sex/age group per 100,000 in the same race or race and sex/age group.

** A total of 681 cases of COVID-19 were reported among 6,598 AI/AN persons aged ≥65 years, and 6,388 cases were reported among 71,395 AI/AN persons aged <65 years.

††
https://svi.cdc.gov/map.html

§§
https://dphhs.mt.gov/Portals/85/ahealthiermontana/2017SHAFinal.pdf

¶¶ The MDPHHS calculated age-adjusted COVID-19 mortality rate for AI/AN persons (374 deaths per 100,000; 95% CI = 322–433) was 7.8 times (95% CI = 6.7–9.1) that among White persons (48 deaths per 100,000; 95% CI = 44–52). Rates were age-adjusted to the 2000 U.S. standard population.
